# Three dimensional evaluation of posture in standing with the PosturePrint: an intra- and inter-examiner reliability study

**DOI:** 10.1186/1746-1340-15-15

**Published:** 2007-09-24

**Authors:** Martin C Normand, Martin Descarreaux, Donald D Harrison, Deed E Harrison, Denise L Perron, Joseph R Ferrantelli, Tadeusz J Janik

**Affiliations:** 1 Département de chiropratique, Université du Québec à Trois-Rivières, Trois Rivieres, Québec, G9A 5H7, Canada; 2 Private Practice, Elko, Nevada, USA; 3 Private Practice, Montréal, Québec, Canada; 4 Private Practice, New Port Richey, Florida, USA; 5 Comp Math R/C, 121 Todd Whitt, Huntsville, AL 35806, USA

## Abstract

**Background:**

Few digitizers can measure the complexity of upright human postural displacements in six degrees of freedom of the head, rib cage, and pelvis.

**Methods:**

In a University laboratory, three examiners performed delayed repeated postural measurements on forty subjects over two days. Three digital photographs (left lateral, AP, right lateral) of each of 40 volunteer participants were obtained, twice, by three examiners. Examiners placed 13 markers on the subjects before photography and chose 16 points on the photographic images. Using the PosturePrint^® ^internet computer system, head, rib cage, and pelvic postures were calculated as rotations (Rx, Ry, Rz) in degrees and translations (Tx, Tz) in millimeters. For reliability, two different types (liberal = ICC_3,1 _& conservative = ICC_2,1_) of inter- and intra-examiner correlation coefficients (ICC) were calculated. Standard error of measurements (SEM) and mean absolute differences within and between observers' measurements were also determined.

**Results:**

All of the "liberal" ICCs were in the excellent range (> 0.84). For the more "conservative" type ICCs, four Inter-examiner ICCs were in the interval (0.5–0.6), 10 ICCs were in the interval (0.61–0.74), and the remainder were greater than 0.75. SEMs were 2.7° or less for all rotations and 5.9 mm or less for all translations. Mean absolute differences within examiners and between examiners were 3.5° or less for all rotations and 8.4 mm or less for all translations.

**Conclusion:**

For the PosturePrint^® ^system, the combined inter-examiner and intra-examiner correlation coefficients were in the good (14/44) and excellent (30/44) ranges. SEMs and mean absolute differences within and between examiners' measurements were small. Thus, this posture digitizer is reliable for clinical use.

## Background

Guidelines for evidence based care include postural evaluation as a primary physical examination procedure to be performed on presenting patients [1,2]. For example, in the fifth edition of the AMA guidelines, standing posture evaluation is recommended as part of a comprehensive but focused spine-related physical examination of the cervical, thoracic, and lumbar spines [1].

Regarding postural measurement methods, there are several tools available for clinical use. These include simple photographic techniques and plumbline measures [3-6], simple goniometers, inclinometers and linear devices [7-9], placing known sized blocks between postural regions [8], various computer assisted methods including electrogoniometers [10], electromagnetic movement systems [11,12], computer assisted digitization systems [13-15], and 3D ultrasound-based motion analysis device for the cervical spine [16].

In today's evidence based health care arena, it is unacceptable to evaluate patients with non-objective measures. Computerized postural digitization/assessment procedures should allow for accurate quantitative evaluation of postural impairments so that improvement or worsening of a patient's abnormality can be succinctly documented.

Validity and reliability of each particular device/system needs to be studied. Several computer assisted postural measurement systems have been studied for measurement reliability [10-16]. Problematically, several of these investigations have suffered from an incomplete analysis, including use of only one examiner, small sample sizes, and measurement of only one region of the body, or a limited number of degrees of freedom of postural displacements [10-16].

Recently, a computerized system, PosturePrint^®^, was developed to measure head, rib cage, and pelvic postures as rotations and translations in three-dimensions (3-D) in upright stance. In two separate validity studies, the PosturePrint^® ^system was found to be sufficiently accurate in measuring head and thoracic cage postures in five degrees of freedom [17,18].

It is the purpose of the present study to evaluate the intra and inter-examiner reliability of the process required for the PosturePrint^® ^computer system's analysis of upright human posture. It was hypothesized that the PosturePrint^® ^would be sufficiently reliable for postural measurements in the clinical setting.

## Methods

### Subjects

Forty student participants underwent a posture evaluation by three examiners, randomly, once each on consecutive days in a University laboratory. The examiners had used the PosturePrint^® ^system previously in their own practices for at least six months. They were not privy to the results of each other's measurements. The study was approved by the Ethics Committee at the University of Quebec in Trois Rivieres, Canada. Participants reviewed the approved Institutional Review Board (IRB) study protocol, provided informed consent for their participation, and their rights were protected.

Since we desired to determine the health status of our participants, participants filled out a four-part Numerical Rating Scale (NRS) of 0–10, (with 0 being no pain and 10 being severe pain), and an SF-36 health questionnaire.

### Study protocol

The posture analysis was performed with the PosturePrint^® ^computer system. The PosturePrint^® ^computer system requires a set of three photographs of each participant: left lateral, antero-posterior (AP), and right lateral. Photographs are obtained with a digital camera. The camera height is at 83.8 cm (33 inches) above the floor and the camera is placed 2.74 m (9 feet) from a calibrated wall grid on a perpendicular line from mid-wall grid. Three such camera and wall grid stations were set up in the University's laboratory with partitions between stations.

Participants stood 61 cm (two feet) from the center of the wall grid. In the AP view, along a line perpendicular from the center of the wall grid, participants positioned their feet such that the perpendicular bisected mid stance. While setting up the wall grid and camera, from mid wall grid, a perpendicular was drawn on the floor outward for 2.74 m. The camera was placed on this line. In each of the two lateral views, the participants' ankles were placed such that the mid ankle bisection was directly inline with the perpendicular from mid wall grid. In this manner, each participant was positioned with their feet centered relative to the camera and grid reference frame.

**Figure 1:**
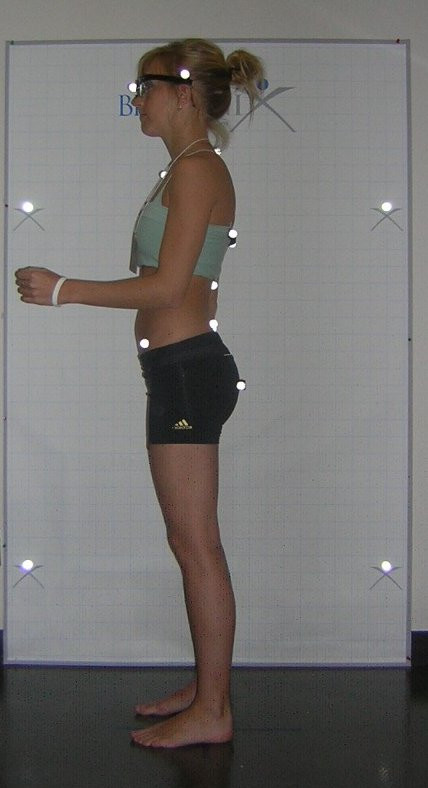
**Participant with markers before photography**. This figure shows one of the three photographs (left lateral view) used in the setting used for this posture reliability study in a university laboratory.

**Figure 2:**
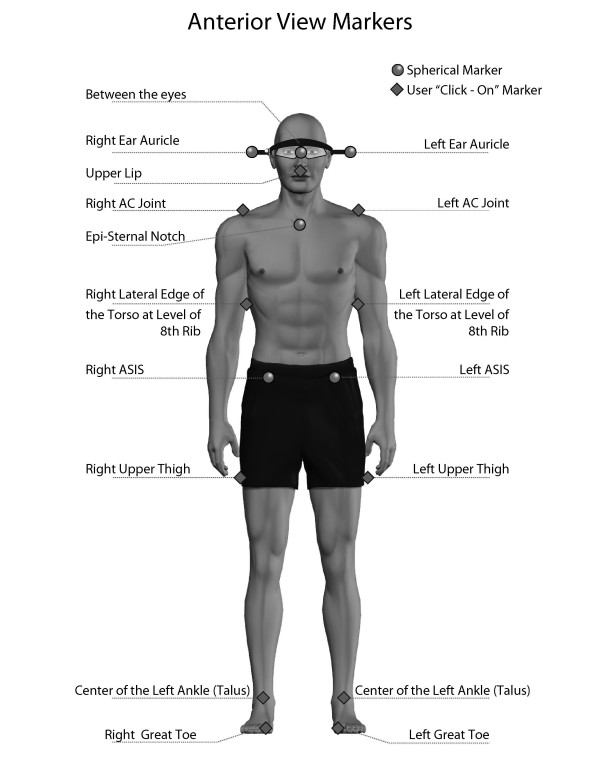
**AP view anatomical markers**. The examiners placed thirteen reflective markers at anatomical locations, which were automatically recognized by the PosturePrint^® ^computer program, and used the computer mouse to click-on/identify 16 more anatomical points. (Reprinted with permission from Biotonix, Montreal, Quebec, Canada)

**Figure 3:**
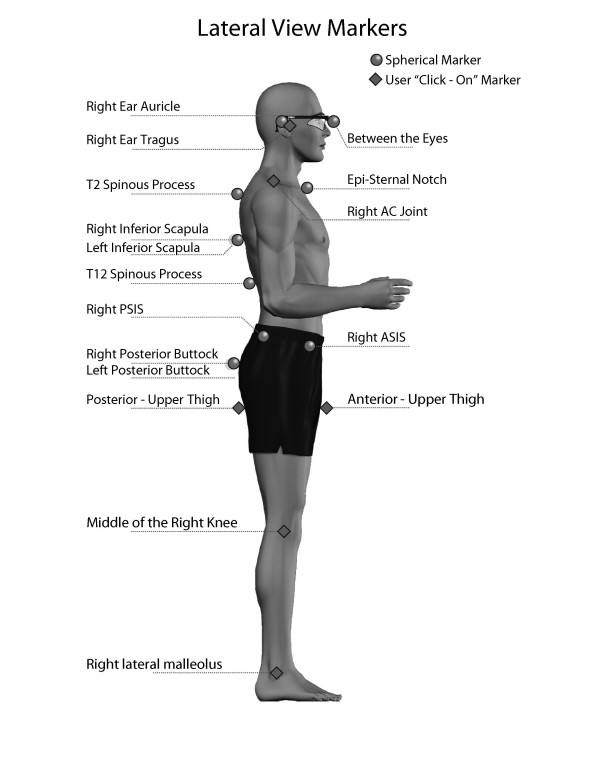
**Lateral view anatomical markers**. The examiners placed 13 reflective markers at anatomical locations, which were automatically recognized by the PosturePrint^® ^computer program, and used the computer mouse to click-on/identify 16 more anatomical points. The computer program calculated postural displacements using these markers. Illustrated here are the markers easily visualized in the lateral view. (Reprinted with permission from Biotonix, Montreal, Quebec, Canada)

The participants were asked to wear tight fitting clothes in order for examiners to find various anatomical sites. The examiners (two of whom are co-authors) placed 13 markers on each participant before taking the three photographs. For the photographs, participants were instructed to stand, nod their head up and down twice with their eyes closed and then assume what they felt to be a neutral body posture. In this stance, the eyes were opened and the subject refrained from motion. (Figure 1) This postural positioning procedure has been shown to be reliable [6,19]. Each examiner palpated anatomical locations and placed his/her own reflective markers, took the three photographs, and removed the markers from each participant before the participant proceeded to the next examiner's camera and wall-grid station. On the digital photographs, using the computer mouse, examiners chose an additional 16 points (Figures 2 and 3). The set of photographs was processed through a secure internet website, where only the coordinates of the markers are available to the computer program (i.e., it is USA HIPAA compliant, Health Insurance Portability and Accountability Act).

All three examiners performed this procedure on all participants twice in two days (80 sets of three photographs). Examiners evaluated twenty participants in two hours during each of two mornings and twenty participants in two hours in the afternoons. Participants were in random order because they were evaluated in the order that they arrived at the beginning station. For each examiner, this resulted in a participant photographic evaluation every 3–6 minutes. Each examiner was given a computer disk with his/her 80 sets of photographs, numbered to blind examiners from names, occasions, and patient characteristics, and was asked to evaluate these on the web site featuring the PosturePrint^® ^over the next two weeks. The data was stored on the web site and accessed by the lead investigator.

**Figure 4:**
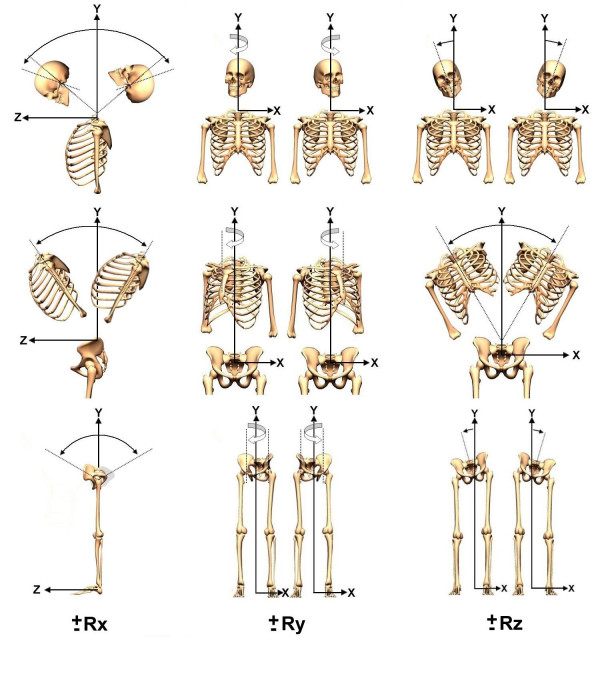
**Postural rotations**. Using a right-handed Cartesian coordinate system with X-axis positive to the left, Y-axis positive vertically, and Z-axis positive to the anterior, postures of the head, rib cage, and pelvis can be described as rotations (Rx, Ry, Rz) around these axes.

**Figure 5:**
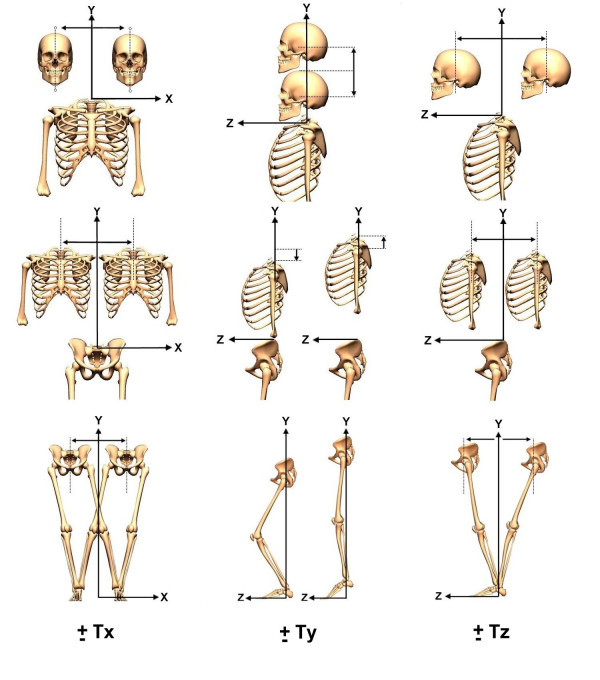
**Postural translations**. Using a right-handed Cartesian coordinate system with X-axis positive to the left, Y-axis positive vertically, and Z-axis positive to the anterior, postures of the head, rib cage, and pelvis can be described as translations (Tx, Ty, Tz) along these axes. Vertical translations (Ty), which would require radiographic analysis of hypo- or hyper-lordosis, were not calculated in the present study.

Using the (x, y)-coordinates and (y, z)-coordinates from the markers on the photographs, the PosturePrint^® ^computer code calculates the static postures of the head, rib cage, and pelvis as rotations (Rx, Ry, Rz) in degrees and translations (Tx, Tz) in millimeters as displacements from a normal upright stance (Figures 4 and 5). Vertical translation (Ty) is not calculated since the center of mass (COM) can not move vertically in static stance.

### Examiner error analysis

If we denote by Y_ijk _the observation on participant i (i = 1,..., 40) by examiner j (j = 1,2,3) on day k (k = 1,2), then for a first error analysis, mean absolute values of the differences within examiners' measurements (MADOM) were calculated as:

MADOM=(∑i=140∑j=13|Yij1−Yij2|)/120.
 MathType@MTEF@5@5@+=feaafiart1ev1aaatCvAUfKttLearuWrP9MDH5MBPbIqV92AaeXatLxBI9gBaebbnrfifHhDYfgasaacH8akY=wiFfYdH8Gipec8Eeeu0xXdbba9frFj0=OqFfea0dXdd9vqai=hGuQ8kuc9pgc9s8qqaq=dirpe0xb9q8qiLsFr0=vr0=vr0dc8meaabaqaciaacaGaaeqabaqabeGadaaakeaacqqGnbqtcqqGbbqqcqqGebarcqqGpbWtcqqGnbqtcqGH9aqpdaqadaqaamaaqadabaWaaabmaeaadaabdaqaaiabdMfaznaaBaaaleaacqWGPbqAcqWGQbGAcqaIXaqmaeqaaOGaeyOeI0IaemywaK1aaSbaaSqaaiabdMgaPjabdQgaQjabikdaYaqabaaakiaawEa7caGLiWoaaSqaaiabdQgaQjabg2da9iabigdaXaqaaiabiodaZaqdcqGHris5aaWcbaGaemyAaKMaeyypa0JaeGymaedabaGaeGinaqJaeGimaadaniabggHiLdaakiaawIcacaGLPaaacqGGVaWlcqaIXaqmcqaIYaGmcqaIWaamcqGGUaGlaaa@5521@

For a second more conservative error analysis, mean absolute values of the differences between examiners' measurements (MADBO) were calculated as:

MADBO=(∑i=140∑j≠j′=13∑k.k′=12|Yijk−Yij′k′|)/480.
 MathType@MTEF@5@5@+=feaafiart1ev1aaatCvAUfKttLearuWrP9MDH5MBPbIqV92AaeXatLxBI9gBaebbnrfifHhDYfgasaacH8akY=wiFfYdH8Gipec8Eeeu0xXdbba9frFj0=OqFfea0dXdd9vqai=hGuQ8kuc9pgc9s8qqaq=dirpe0xb9q8qiLsFr0=vr0=vr0dc8meaabaqaciaacaGaaeqabaqabeGadaaakeaacqqGnbqtcqqGbbqqcqqGebarcqqGcbGqcqqGpbWtcqGH9aqpdaqadaqaamaaqadabaWaaabmaeaadaaeWaqaamaaemaabaGaemywaK1aaSbaaSqaaiabdMgaPjabdQgaQjabdUgaRbqabaGccqGHsislcqWGzbqwdaWgaaWcbaGaemyAaKMafmOAaOMbauaacuWGRbWAgaqbaaqabaaakiaawEa7caGLiWoaaSqaaiabdUgaRjabc6caUiqbdUgaRzaafaGaeyypa0JaeGymaedabaGaeGOmaidaniabggHiLdaaleaacqWGQbGAcqGHGjsUcuWGQbGAgaqbaiabg2da9iabigdaXaqaaiabiodaZaqdcqGHris5aaWcbaGaemyAaKMaeyypa0JaeGymaedabaGaeGinaqJaeGimaadaniabggHiLdaakiaawIcacaGLPaaacqGGVaWlcqaI0aancqaI4aaocqaIWaamcqGGUaGlaaa@61D9@

### Intraclass correlation coefficients

Our methodology provided three sets of intra-examiner data and three sets of inter-examiner data. Inter- and intraclass correlation coefficients (ICCs) were calculated for each of 15 variables (measurement type). We provided two different methods of calculating ICCs, Standard Errors of Measurement (SEM), and Differences in Examiner's Measurements for a conservative (ICC_2,1_) and a more usual (liberal, ICC_3,1_) analysis of our data. The use of ICCs require a normal distribution of the data, which was determined with the Shapiro-Wilk test.

For the ICC_2,1 _method, all statistical calculations were done using SAS 9.0 (SAS Institute, Inc. Cary, NC, USA). For the liberal method (ICC_3,1_), ANOVAs were computed using Statistica for windows (2001) Version 6.0 (Statsoft, Tulsa, OK, USA).

### Standard error of measurement (SEM)

According to Weir [20], ICCs are a relative measure of reliability, while the Standard Error of Measurement (SEM) provides an absolute index of reliability. Therefore, SEMs were calculated for both ICC methods, of which the conservative method has the lowest ICCs and highest SEMs. If SD represents the standard deviation of the scores from all subjects, then from Weir [20]:

SEM=SD⋅1−ICC.
 MathType@MTEF@5@5@+=feaafiart1ev1aaatCvAUfKttLearuWrP9MDH5MBPbIqV92AaeXatLxBI9gBaebbnrfifHhDYfgasaacH8akY=wiFfYdH8Gipec8Eeeu0xXdbba9frFj0=OqFfea0dXdd9vqai=hGuQ8kuc9pgc9s8qqaq=dirpe0xb9q8qiLsFr0=vr0=vr0dc8meaabaqaciaacaGaaeqabaqabeGadaaakeaacqqGtbWucqqGfbqrcqqGnbqtcqGH9aqpcqqGtbWucqqGebarcqGHflY1daGcaaqaaiabigdaXiabgkHiTiabbMeajjabboeadjabboeadbWcbeaakiabc6caUaaa@3BB0@

## Results

The participants were composed of 30 females and 10 males, with a mean age of 24.4 years (SD = 1.9). Their mean height was 168.8 cm (SD = 8.5) and mean weight was 62.5 kg (SD = 11.2). The four-part numerical rating scores (NRS) for pain and Short Form questionnaire (SF-36) scores indicated a near normal group (Tables 1 and 2).

**Table 1:** Numerical Rating Scale (NRS:0,1,..., 10) for pain in the forty volunteer subjects at different times during a typical day.

	NRS Now	NRS Average	NRS Best	NRS Worst
Mean ± Standard deviation	1.0 ± 1.3	1.1 ± 1.7	0.05 ± 0.2	3.5 ± 3.2

**Table 2:** The Short Form questionnaire (SF36) for activities of daily living in the forty volunteer subjects

	health perception	physical functioning	role physical	role emotional	social functioning	mental health	bodily pain	energy fatigue
Mean ± Standard Deviation	88.4 ± 7.5	98.9 ± 3.1	95.0 ± 18.1	80.8 ± 35.3	83.9 ± 19.6	71.3 ± 14.8	84.6 ± 14.3	62.0 ± 16.7

We noted that for four variables (RyHead, RyThorax, RyPelvis, and RzPelvis), the assumption of having a normal distribution was violated. For these variables, values were closely distributed with many identical values making it inappropriate to calculate ICCs. In such cases, examiner agreement is high but inappropriately calculated ICCs may be artificially low [20]. For such situations, it is more informative to consider the SEM [20]. For these four variables, the more conservative SEMs are 0.9°, 1.2°, 1.2°, and, 1.3°, respectively, which indicates excellent reliability. Table 3 provides SEMs for all fifteen variables for the conservative ICC method. These SEMs are 2.7° or less for all rotations and 5.9 mm (approximately 1/4 inch) or less for all translations.

**Table 3:** PosturePrint^® ^reliability analysis with a conservative ICC method (ICC_2,1_). Three examiners evaluated forty student volunteers, with a posture digitizer, twice over 2 days.

**Measure**	**Mean**	SD*	**SEM†**	**InterICC‡**	**95% C.I. §**	**Intra ICC‡**	**95% C.I.**	**MADBO||**
Pelvis Rx	3.2°	4.5	2.4°	0.56	0.44–0.68	0.72	0.59 – 0.82	3.3° ± 2.6
Thorax Rx	-3.1°	3.1	1.5°	0.65	0.53–0.76	0.76	0.64 – 0.86	2.3° ± 1.7
Head Rx	-1.2°	5.7	2.7°	0.69	0.58–0.79	0.77	0.66 – 0.86	3.5° ± 2.7
Pelvis Ry	-0.3°	1.1	0.9°	N/A¶	N/A	N/A	N/A	0.6° ± 1.2
Thorax Ry	0.2°	1.3	1.2°	N/A	N/A	N/A	N/A	1.0° ± 1.4
Head Ry	-0.1°	1.8	1.2°	N/A	N/A	N/A	N/A	1.6° ± 1.3
Pelvis Rz	-0.3°	1.5	1.3°	N/A	N/A	N/A	N/A	1.5° ± 1.3
Thorax Rz	0.3°	1.4	0.7°	0.66	0.55–0.77	0.76	0.66–0.85	1.0° ± 0.8
Head Rz	1.1°	2.3	1.2°	0.66	0.55–0.77	0.75	0.64–0.84	1.9° ± 1.3
Pelvis Tx	-3.6 mm	7.1	4.3 mm	0.51	0.39 – 0.64	0.64	0.51 – 0.76	6.0 mm ± 4.3
Thorax Tx	2.0 mm	6.5	2.6 mm	0.72	0.62 – 0.82	0.84	0.74 – 0.90	4.2 mm ± 3.2
Head Tx	-0.6 mm	6.0	3.4 mm	0.54	0.42 – 0.67	0.67	0.54 – 0.78	5.2 mm ± 3.9
Pelvis Tz	54.4 mm	17.0	5.9 mm	0.80	0.71 – 0.87	0.88	0.81 – 0.93	8.4 mm ± 6.3
Thorax Tz	3.6 mm	9.8	5.5 mm	0.54	0.41 – 0.68	0.68	0.52 – 0.81	8.3 mm ± 6.0
Head Tz	30.1 mm	9.1	4.6 mm	0.64	0.52 – 0.75	0.75	0.64 – 0.84	6.0 mm ± 4.6

* SD = Standard deviation

† SEM = Standard error of measurement = SD⋅1−ICC
 MathType@MTEF@5@5@+=feaafiart1ev1aaatCvAUfKttLearuWrP9MDH5MBPbIqV92AaeXatLxBI9gBaebbnrfifHhDYfgasaacH8akY=wiFfYdH8Gipec8Eeeu0xXdbba9frFj0=OqFfea0dXdd9vqai=hGuQ8kuc9pgc9s8qqaq=dirpe0xb9q8qiLsFr0=vr0=vr0dc8meaabaqaciaacaGaaeqabaqabeGadaaakeaacqqGtbWucqqGebarcqGHflY1daGcaaqaaiabigdaXiabgkHiTiabbMeajjabboeadjabboeadbWcbeaaaaa@365D@.

‡ ICC = Cross factor intra- or inter-examiner correlation coefficient

§ 95% C.I. = 95 percent confidence interval

|| MADBO = Mean absolute differences between observers' measurements

¶ N/A = Not applicable, means the assumption of a normal distribution was violated by many identical values, and thus ICCs are not appropriate for this data.

### Conservative ICCs

The conservative method of calculating ICCs (ICC_2,1_) often has inter-examiner ICCs lower than other ICC methods by approximately 0.1 [21]. For the 11 variables (out of 15) for which ICCs were appropriate, all the conservative intra- and inter-examiner ICCs were in the good range (0.5 < ICC < 0.75) or excellent range (ICC ≥ 0.75) [22]. In general, the intra-examiner ICCs (seven out of eleven were greater than 0.75) were higher than the inter-examiner ICCs. Only four of the inter-examiner ICCs were in the lower portion of the good range (0.5 < ICC < 0.60) and seven inter-examiner ICCs were greater than 0.60, while one (Tz of the Pelvis) was in the excellent range. For the variables for which these conservative ICC values are in the good range (0.5 < ICC < 0.75), we note that the standard deviations are small (Table 3), indicating participants differ little from each other [20]. For these variables, SEMs are more informative [20]. The small SEMs in Table 3 indicate high reliability. Table 3 provides the details of this conservative analysis with means, standard deviations, SEMs, ICCs, and 95% confidence intervals.

### Liberal ICCs

For the 11 variables (out of 15) for which ICCs were appropriate, all of the intra- and inter-examiner ICCs for this more liberal method (ICC_3,1_) were greater than 0.84, which is in the excellent range as defined by Shrout and Fleiss (ICC ≥ 0.75) [22] and in the almost perfect range as suggested by Dunn (ICC > 0.80) [23]. Table 4 provides SEMs, intra- and inter-examiner correlation coefficients, and 95% confidence intervals.

**Table 4:** PosturePrint^® ^reliability with a liberal ICC method (ICC_3,1_). Forty student volunteers at a university were evaluated, with a posture digitizer, twice by three examiners, with a one-day delay.

**Measure**	SEM*	**InterICC†**	**95% C.I. ‡**	**Intra ICC†**	**95% C.I.**	**MADOM§**
Pelvis Rx	1.3°	0.88	0.81–0.93	0.92	0.80 – 0.94	2.6° ± 2.1
Thorax Rx	0.9°	0.93	0.88–0.96	0.92	0.81 – 0.94	1.6° ± 1.3
Head Rx	1.5°	0.95	0.92–0.97	0.93	0.82 – 0.94	3.2° ± 2.5
Pelvis Ry	0.4°	N/A||	N/A	N/A	N/A	0.6° ± 1.2
Thorax Ry	0.8°	N/A	N/A	N/A	N/A	0.9° ± 1.4
Head Ry	0.6°	N/A	N/A	N/A	N/A	1.1° ± 1.0
Pelvis Rz	0.9°	N/A	N/A	N/A	N/A	1.4° ± 1.4
Thorax Rz	0.4°	0.93	0.89–0.96	0.92	0.79–0.94	0.7° ± 0.6
Head Rz	0.6°	0.94	0.90–0.97	0.94	0.84–0.95	1.1° ± 0.8
Pelvis Tx	1.7 mm	0.89	0.82 – 0.94	0.94	0.85 – 0.96	3.9 mm ± 3.1
Thorax Tx	1.7 mm	0.94	0.90 – 0.97	0.93	0.82 – 0.95	3.3 mm ± 2.5
Head Tx	2.3 mm	0.89	0.83 – 0.94	0.85	0.64 – 0.88	4.0 mm ± 2.8
Pelvis Tz	2.9 mm	0.96	0.94 – 0.98	0.97	0.91 – 0.97	7.1 mm ± 5.6
Thorax Tz	2.9 mm	0.89	0.82 – 0.93	0.91	0.78 – 0.93	5.7 mm ± 4.5
Head Tz	2.6 mm	0.93	0.88 – 0.96	0.92	0.79 – 0.93	5.4 mm ± 4.7

* SEM = Standard error of measurement = SD⋅1−ICC
 MathType@MTEF@5@5@+=feaafiart1ev1aaatCvAUfKttLearuWrP9MDH5MBPbIqV92AaeXatLxBI9gBaebbnrfifHhDYfgasaacH8akY=wiFfYdH8Gipec8Eeeu0xXdbba9frFj0=OqFfea0dXdd9vqai=hGuQ8kuc9pgc9s8qqaq=dirpe0xb9q8qiLsFr0=vr0=vr0dc8meaabaqaciaacaGaaeqabaqabeGadaaakeaacqqGtbWucqqGebarcqGHflY1daGcaaqaaiabigdaXiabgkHiTiabbMeajjabboeadjabboeadbWcbeaaaaa@365D@.

† ICC = Intra- or inter-examiner correlation coefficient

‡ 95% C.I. = 95 percent confidence interval

§ MADOM = Mean absolute differences of observers' measurements

|| N/A = Not applicable, means the assumption of a normal distribution was violated by many identical values, and thus ICCs are not appropriate for this data.

### Error analysis

Two types of error analysis were computed from equations 1 and 2. Since each examiner had two measurements for each variable, three examiners provided three pairs of differences of first and second measurements. The more liberal MADOM averaged the three pairs of differences within the same examiner over all subjects [24].

However, if the first and second measurements for each examiner were not compared to each other, but to each of the measurements of the other examiners, then there were twelve pairs of differences for each variable. The more conservative MADBO error analysis averaged all of the 12 pairs of differences for different examiners over all subjects.

The mean absolute differences between examiners' (MADBO) measurement results were 6 mm (≈ 1/4 inch) or less for lateral translations (TxH, TxT, TxP) and 8.4 mm (1/3 inch) or less for forward translational measurements (TzH, TzT, TzP). The MADBO were 3.5° or less for flexion-extension rotational measurements (RxH, RxT, RxP) and 1.9° or less for all axial rotations (RyH, RyT, RyP) and lateral bending rotations (RzH, RzT, RzP). Table 3 provides all the MADBO results.

The mean absolute differences within examiners' measurements (MADOM) results were 4 mm (≈ 1/6 inch) or less for lateral translations (TxH, TxT, TxP) and 7.1 mm (≈ 1/4 inch) or less for forward translational measurements (TzH, TzT, TzP). The MADOM were 3.2° or less for flexion-extension rotational measurements (RxH, RxT, RxP) and 1.4° or less for all axial rotations (RyH, RyT, RyP) and lateral bending rotations (RzH, RzT, RzP). Table 4 provides the MADOM results.

## Discussion

This study assessed the intra and inter-examiner reliability of standing posture with a new computerized postural digitizer, PosturePrint^®^, using three examiners, who evaluated forty subjects each on two different occasions. It had been hypothesized that the PosturePrint^® ^would be a reliable method to evaluate head, rib cage, and pelvic posture as three rotations and two translations or five degrees of freedom (DoF). In fact for 11 out of 15 variables (a total of 44 Intra- and Inter-examiner ICCs), 14 (32%) were in the good range (0.50 < ICC < 0.75) and 30 (68%) were in the excellent range (ICC > 0.75) [22]. For the four postural variables, for which ICCs were inappropriate, small SEMs (1.3° or less for these axial and lateral flexion rotations) indicate excellent reliability. Additionally for all variables, small SEMs and small mean absolute errors (two types) indicate close examiner agreement. Thus, the data indicate that the PosturePrint^® ^is rated good to excellent for reliability of measuring standing posture.

### Study limitations

One possible limitation of this study might be the fact that our participant population represented a relatively asymptomatic population with an average NRS of 1.1 ± 1.7. However, postural analysis has been shown to be repeatable in a variety of pain populations as well as asymptomatic groups [19]. Some evidence in recent acute whiplash injured subjects suggested that head position sense is not repeatable [25], but certain measures (forward head posture) in this group have been found to be reliable [9].

Sources of error in the PosturePrint^® ^systems' analysis of posture included: possible variation in upright stance from day to day, inherent errors due to placing markers from palpation of boney landmarks [26], errors involved in the choosing of sixteen points on the photographs via the computer mouse by each examiner, and errors in positioning the participants in the same manner relative to the reference wall grid and camera [27]. However, the high ICCs, small SEMs, and low mean absolute differences between and within examiners' measurements indicate that these sources of error were kept at a minimum.

Another limitation might be the choice of the ICC method used [13,20,28,29]. The definition of the ICC method depended on the assumptions of (a) whether each of examiners, time, and participants was a fixed or random factor and (b) the type of error included (true score variance, systematic and/or random) [28]. In the equation for calculating ICCs, this changed the denominator [20]. For the conservative method, it was assumed that measurement was crossed with examiner and participant, and examiner, participant, and occasion were all random factors. This enlarged the denominator in the definitions of the conservative ICCs, making ICCs smaller. Additionally, the magnitude of an ICC depended on the between-participants variability [20]. By providing both a liberal and conservative methods and SEMs for each method, we have reduced any limitations due to choice of an ICC method.

Depending on the ICC type of equations used, between 30 and 60 participants would be necessary for a conclusion of reliability to be made [21,30]. Estimations from Eliasziw et al. [29] suggest that for 0.9 reliability and two repeated measurements, 40 participants were more than adequate for a 5% significance with 80% power. Because of this, the current investigators used 40 participants with three examiners assessing each participant twice with a one day interval between measurements.

According to Weir [20], "there are six common versions of the ICC (and four others as well), and the choice of which version to use is not intuitively obvious." Additionally, there are 10 ICC versions presented by McGraw and Wong [28]. This is the reason why we decided to report two types of ICCs to be calculated for each of fifteen variables, a more liberal method and a more conservative method.

The two sets of ICCs were calculated under slightly different tenable model assumptions. For the conservative type, measurement was crossed with examiner and participant, and examiner was a random factor [21]. Results from this type of ICC (a generalization of ICC_2,1_) can be generalized to subject and examiner populations [20].

The liberal ICC method assumed that the three factors (examiner, participant, occasion) were fixed and used a two-way repeated-measures ANOVA model. Two-way ICC models (this liberal ICC type is ICC_3,1_) required occasions or examiners to be crossed with participants (i.e., each examiner evaluated all participants on each occasion in the present study) [20]. Use of this ICC type restricted how the results can be generalized. However, it can be used to identify the limits and pitfalls of postural analysis (e.g.: marker placement).

Therefore, the denominator in the equation to compute the liberal ICCs were the sum of two terms, while the denominators of the conservative ICCs were the sum of three terms, which makes the conservative ICCs smaller than the liberal ICCs.

### Previous studies

A few studies have investigated the repeatability of postural measures using computer assisted devices [10-16]. Some studies did not report reliability in terms of ICCs [10,15]. However, we noted that some studies have reported small ICCs and claimed poor posture reliability [12-15], when in fact, their data suggested that ICCs were inappropriate for certain variables. According to Weir [20], there were at least two instances when ICCs are not informative: (a) when multiple repeated measured values occur in the data and (b) when data is homogenous. ICCs, of any type, should not be used on measurements that are mostly one value because this violated a basic ANOVA assumption that the data were approximately normally distributed. This meant that the data must be spread out over a continuum, with concentration in the middle and symmetry about the middle. If there was a normal distribution, but the distribution had a very small standard deviation, then Weir stated [20], "if subjects differ little from each other, ICC values are small even if trial-to-trial variability is small." Weir's ideas may apply to two recent studies by Dunk et al [13,14].

Dunk et al. [13] performed a reliability study of a photographic technique and consequent digitization of reflective landmarks with 14 participants and reported poor to moderate ICCs for posture reliability. After a letter to the editor [27] critical of their 2004 study [13], in a follow up study [14], Dunk et al. assessed the intra-examiner reliability with more (20) healthy participants. Dunk et al. concluded that their sagittal plane measures were more reliable than coronal plane measures, but their sagittal plane angles of spinal curvature had mean error of approximately 6° while their coronal plane bending had mean error less than 2° [14]. Because Dunk et al. [14] had an error of 6° with high ICCs for the sagittal plane, but a very small error of 2° with low ICCs for the coronal plane, it may be that either multiple values occurred in Dunk et al's data or their participants were quite similar. Thus, their conclusions of poor reliability for coronal plane bending may be incorrect.

Using an electromagnetic device, Swinkles and Dolan examined the ability of healthy individuals to reposition their thoraco-lumbar regions in both sagittal and coronal planes (two DoF) [11,12]. Intra-day and inter-day repeated measures were found to be 5° or less for sagittal displacements and 2.5° or less for coronal displacements. Although, Swinkles and Dolan [11,12] found some ICCs to be in the poor range, they commented that several of their displacement values were very small and approached the limit of accuracy of their measurement device. Here, the use of ICCs on these variables was inappropriate as explained above (see Weir [20]). Consequently, considering the small repositioning errors, they concluded that "healthy volunteers were able to reposition their spine with considerable accuracy as measured with the 3-Space Fastrak" [11].

In another reliability study of posture, using an ultrasonic digitizer (Zebris) method of cervical range of motion measurements, Strimpakos et al. stated that their method employed for measuring cervical joint position sense was unreliable [16].

### Posture reliability design suggestions

According to the above review, there were a variety of methodological concerns with reported reliability studies in the literature. For example, many investigations utilized only one examiner and it was possible that this examiner could have made gross mistakes from one examination to the next, causing poor intra-examiner reliability. Statistically, therefore, multiple examiners were needed to average any artificially low or high intra-examiner data, which would provide a more reasonable mean. It has been suggested that a minimum of three examiners each performing an analysis at least twice was needed for any conclusions to be drawn about inter- and intra-examiner reliability [30]. In the current investigation of the PosturePrint^® ^system, we have followed this recommendation.

Lastly, depending upon the mean value and distribution of the specific postural displacement recorded, ICCs may be inappropriate as they cannot give a clinically relevant picture of the true error. Because of this, in the current investigation, we analyzed the Standard Error of Measurement (SEM) and mean absolute differences within (MADOM) and between (MADBO) examiners' measurements for each postural degree of freedom. The SEMs were small (2.7° or less for all rotations and 5.9 mm (≈ 1/4 inch) or less for all translations). The MADOM values were found to be 4 mm or less for lateral translations and 7.1 mm or less for forward translations. The MADOM values were 3.2° or less for flexion-extension rotational measurements and 1.4° or less for all axial rotations and lateral bending rotations. The MADBO values were found to be 6 mm or less for lateral translations and 8.4 mm or less for forward translations. The MADBO values were 3.5° or less for flexion-extension rotational measurements and 1.9° or less for all axial rotations and lateral bending rotations.

Since the PosturePrint^® ^system has adequate reliability, there were several possible future studies. A study on healthy subjects could provide a normative database. Studies on patients could provide any differences from normal. Correlations between different postures and health conditions are possible, and pre- and post-treatment clinical trials with various technique methods are also possible studies.

## Conclusion

When three examiners evaluated 40 participants twice with a posture digitizer, the PosturePrint^®^, it was found to be highly reliable. The SEMs were small, observers' errors were small, and the combined 44 inter-examiner and intra-examiner correlation coefficients were in the good (14/44) and excellent (30/44) ranges for clinical research. Thus, the PosturePrint^® ^computer system was determined to be highly reliable for measuring neutral standing posture.

## Competing interests

Six authors declare that they have no competing interests, while one author (DDH) has a financial agreement with Biotonix, which provides the web service for PosturePrint.

## Authors' contributions

All authors contributed to the research design (MN, MD, DDH, DEH, DP, JF, TJ). MN, DDH, DP, and JF collected the data. MD and TJ analyzed the data. All authors participated in drafting the manuscript. All authors read and approved the final manuscript
